# Practical Recommendations for the Diagnosis and Management of Lysosomal Acid Lipase Deficiency with a Focus on Wolman Disease

**DOI:** 10.3390/nu16244309

**Published:** 2024-12-13

**Authors:** Javier de las Heras, Carolina Almohalla, Javier Blasco-Alonso, Mafalda Bourbon, Maria-Luz Couce, María José de Castro López, Mª Concepción García Jiménez, David Gil Ortega, Luisa González-Diéguez, Silvia Meavilla, Ana Moreno-Álvarez, José Pastor-Rosado, Paula Sánchez-Pintos, Irene Serrano-Gonzalo, Eduardo López, Pedro Valdivielso, Raquel Yahyaoui, Jesús Quintero

**Affiliations:** 1 Division of Pediatric Metabolism, Cruces University Hospital, CIBER-ER, Metab-ERN, University of the Basque Country (UPV/EHU), Biobizkaia Health Research Institute, 48903 Bilbao, Spain; 2 Unidad de Hepatología, Hospital Universitario Río Hortega, 47012 Valladolid, Spain; calmohallaalvareza@saludcastillayleon.es; 3 Unidad de Diagnóstico y Tratamiento de Enfermedades Metabólicas Hereditarias, UGC Pediatría, Hospital Regional Universitario de Málaga, 29010 Málaga, Spain; javierblascoalonso@yahoo.es; 4 Grupo de Investigação Cardiovascular, Departamento de Promoção da Saúde e Prevenção de Doenças não Transmissíveis, Instituto Nacional de Saúde Doutor Ricardo Jorge, 1649-016 Lisboa, Portugal; mafalda.bourbon@insa.min-saude.pt; 5 BioISI, Biosystems & Integrative Sciences Institute, Faculdade de Ciências, Universidade de Lisboa, 1649-004 Lisboa, Portugal; 6 Metabolic Unit, Department of Forensic Sciences, Pathology, Gynecology and Obstetrics, Pediatrics, Health Research Institute of Santiago de Compostela (IDIS), Hospital Clínico Universitario de Santiago de Compostela, CIBERER, MetabERN, 15706 Santiago de Compostela, Spain; maria.luz.couce.pico@sergas.es (M.-L.C.); paula.sanchez.pintos@sergas.es (P.S.-P.); 7 Willink Biochemical Genetics Unit, St Mary’s Hospital, Manchester University Foundation Trust, University of Manchester, Manchester M13 9WL, UK; maria.lopez@mft.nhs.uk; 8 NeuroMetabolic Unit, Pediatría, Hospital Universitario Miguel Servet, Aragon Health Research Institute (IIS Aragón), 50009 Zaragoza, Spain; igarciaji@salud.aragon.es; 9 Unidad de Gastroenterología, Hepatología y Nutrición Pediátrica, Hospital Universitario Virgen de la Arrixaca, 30120 Murcia, Spain; dr.gil.ortega@gmail.com; 10 Liver Unit, Division of Gastroenterology and Hepatology, Hospital Universitario Central de Asturias, 33011 Oviedo, Spain; luisagondi@hotmail.com; 11 Metabolic Unit, Gastroenterology, Hepatology and Nutrition Department, Sant Joan de Déu Hospital, 08950 Barcelona, Spain; silviamaria.meavilla@sjd.es; 12 Pediatric Gastroenterology, Hepatology and Nutrition Unit, Department of Pediatrics, A Coruña University Hospital, 15006 A Coruña, Spain; ana.moreno.alvarez@sergas.es; 13 Lipid Unit, Department of Pediatrics, Hospital General Universitario de Elche, Universidad Miguel Hernandez de Elche, 03202 Elche, Spain; pastor_jos@gva.es; 14 Fundación Española Para el Estudio y Terapéutica de la Enfermedad de Gaucher y Otras Lisosomales (FEETEG), 50009 Zaragoza, Spain; ireneserranogonzalo@gmail.com; 15 GIIS-012 Group, Aragon Health Research Institute (IIS Aragón), 50009 Zaragoza, Spain; 16 Spanish LAL-D Patient Organization, 08918 Badalona, Spain; eduardolopezsantamaria36@gmail.com; 17 Unidad de Lípidos, Hospital Universitario Virgen de la Victoria, Instituto de Investigación Biomédica de Málaga (IBIMA), Universidad de Málaga, 29010 Málaga, Spain; valdivielso@uma.es; 18 Clinical Laboratory, Laboratory of Inherited Metabolic Disorders, Hospital Regional Universitario de Málaga, Instituto de Investigación Biomédica de Málaga IBIMA-Plataforma BIONAND, 29590 Málaga, Spain; raquelyahyaoui@gmail.com; 19 Pediatric Hepatology and Liver Transplant Unit, Department of Pediatrics, ERN Rare Liver ERN TransplantChild, Vall d’Hebron Barcelona Hospital Campus, Universitat Autònoma de Barcelona, 08193 Barcelona, Spain; jesus.quintero@vallhebron.cat

**Keywords:** LAL-D, lysosomal acid lipase deficiency, infantile-onset lysosomal acid lipase deficiency, Wolman disease, cholesteryl ester storage disease, diet, enzyme replacement therapy, *LIPA* gene, lysosomal acid lipase, sebelipase

## Abstract

Lysosomal acid lipase deficiency (LAL-D) is an ultra-rare lysosomal storage disease with two distinct phenotypes, an infantile-onset form (formerly Wolman disease) and a later-onset form (formerly cholesteryl ester storage disease). The objective of this narrative review is to examine the most important aspects of the diagnosis and treatment of LAL-D and to provide practical expert recommendations. The infantile-onset form occurs in the first weeks of life and is characterized by malnourishment and failure to thrive due to gastrointestinal impairment (vomiting, diarrhea, malabsorption), as well as systemic inflammation, hepatosplenomegaly, and adrenal calcifications. Mortality is close to 100% before one year of life in the absence of specific treatment. The later-onset form can be diagnosed in childhood or adulthood and is characterized by chronic liver injury and/or lipid profile alterations. When LAL-D is suspected, enzyme activity should be determined to confirm the diagnosis, with analysis from a dried blood spot sample being the quickest and most reliable method. In infantile-onset LAL-D, the initiation of enzyme replacement therapy (sebelipase α) and careful nutritional management with a low-lipid diet is very urgent, as prognosis is directly linked to the early initiation of specific treatment. In recent years, our knowledge of the management of LAL-D has increased considerably, with improvements regarding the initial enzyme replacement therapy dose and careful nutritional treatment with a low-lipid diet to decrease lipid deposition and systemic inflammation, leading to better outcomes. In this narrative review we offer a quick guide for the initial management of infantile-onset LAL-D.

## 1. Introduction

Lysosomal acid lipase deficiency (LAL-D; OMIM 278000) is a severe autosomal recessive disorder involving multiple organs [1]. It is caused by biallelic pathogenic variants of the *LIPA* gene (MIM*613497) that cause decreased lysosomal acid lipase (LAL) activity [2]. LAL catalyzes the intralysosomal hydrolysis of the cholesterol esters and triglycerides contained in low-density lipoprotein (LDL). A decrease or absence in the activity of LAL impairs metabolism, leading to the accumulation of these lipids in the lysosomes where the LDL receptors are located, especially in the liver and in the cells of the mononuclear phagocyte system [3,4]. These lysosomal deposits give rise to different clinical manifestations owing to abnormal lipid storage in the liver, spleen, and gastrointestinal tract [1,2,5]. Diagnosis is confirmed via significantly decreased LAL activity in cultured skin fibroblasts, leukocytes, and dried blood spot samples or by identification of biallelic pathogenic *LIPA* variants.

LAL-D affects individuals from birth to adulthood. Its broad phenotypic spectrum is characterized by two different phenotypes. At one end of the spectrum is the infantile-onset form (or Wolman disease), which is characterized by a complete loss or less than 1% of normal enzyme activity [2]. Infantile-onset disease has a rapidly progressive and severe course and is diagnosed in neonates and infants, with a prevalence of approximately 1 per 350,000 live births [3,6]. At the other end of the spectrum is the later-onset form (or cholesteryl ester storage disease), which is characterized by significantly reduced enzyme activity, typically 1% to 12% of normal activity [2]. Later-onset disease is diagnosed mainly in children and adults, with a prevalence of approximately 1 per 300,000 individuals [1,3,6]. The rapidly progressive infantile-onset form is particularly severe, with high mortality if appropriate treatment is not initiated early. In the absence of early treatment, the median age at death is 3.7 months (1.4–46.3 months) [3]. Although the later-onset form is less severe, it can lead to hepatic fibrosis/cirrhosis, accelerated atherosclerosis, premature cardiovascular disease, and early death [1,2,3,7]. Despite the rarity of LAL-D, the genetic prevalence of known *LIPA* variants predicts a higher incidence of the disease than reported, indicating that it is under-diagnosed [2,8].

Survival of rapidly progressive infantile-onset LAL-D has been found to improve dramatically with enzyme replacement therapy (ERT) [7]. However, mortality was found to remain rather high in the clinical trials, as patients developed severe complications, caused mainly by malabsorption because of gut damage and systemic inflammation [9]. In recent years, our knowledge of the management of these patients has increased considerably, with improvements regarding the initial ERT dose and careful nutritional treatment to decrease systemic inflammation leading to better outcomes in the real world.

The medical and nutritional management of infantile-onset LAL-D can be challenging. To our knowledge, there are no published data to guide physicians in the early stage, when fatal disease is most frequent.

Thus, the objectives of the current narrative review are as follows: (1) to examine the most important aspects of the diagnosis, treatment, and follow-up of LAL-D, including expert recommendations and (2) to provide simple and practical recommendations that will help clinicians to manage this rare disease by initiating treatment as early as possible to thus improve prognosis.

## 2. Methods

This narrative review was performed following three steps: a search of the literature, a review of the abstracts and full texts, and a discussion of the information provided in the articles. The PubMed and Google Scholar databases were used for the bibliographic search, with the following keywords: “LAL-D”, “lysosomal acid lipase deficiency”, “infantile-onset lysosomal acid lipase deficiency”, “Wolman disease”, “cholesteryl ester storage disease”, “diet”, “enzyme replacement therapy”, “LIPA gene”, “lysosomal acid lipase”, and “sebelipase”. After the complete search, the abstracts were read to ensure that they addressed the topic of interest. As this is a narrative review, it was unnecessary to document the literature search on specific platforms.

As LAL-D is an ultra-rare disease, most of the practical information provided in this manuscript is based on expert recommendations. The drafting of this narrative review involved pediatricians and adult physicians with experience treating patients affected by LAL-D (both the infantile-onset and the later-onset phenotypes), clinical laboratory specialists knowledgeable in the condition, and the president of the Spanish LAL-D Patient Organization.

## 3. Clinical Manifestations

### 3.1. Infantile-Onset LAL-D (Wolman Disease)

**Figure 1:**
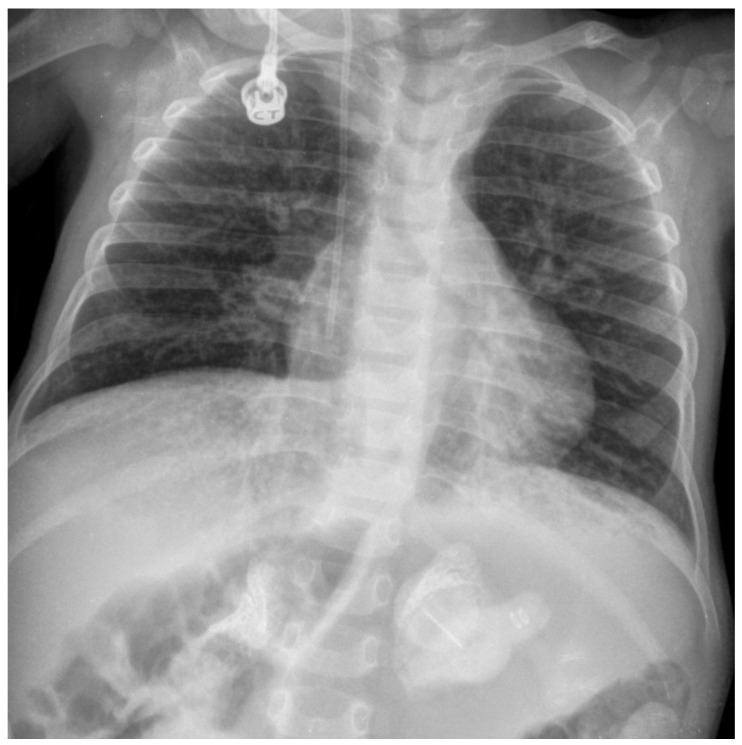
Abdominal X-ray showing adrenal gland calcifications in a patient with infantile-onset lysosomal acid lipase deficiency (LAL-D). Image courtesy of Dr. de las Heras.

The onset of symptoms is during the first weeks of life, usually before the age of one month. The main clinical manifestations of infantile-onset LAL-D include digestive disorders, such as malabsorption, steatorrhea, vomiting, and diarrhea, leading to malnourishment and failure to thrive, as well as persistent abdominal distension with hepatosplenomegaly (Table 1) [10]. The presence of adrenal calcifications (Figure 1) strongly suggests LAL-D, although their absence does not rule out the disease, particularly in the early stages. These calcifications can be detected using imaging methods, such as abdominal X-ray, ultrasound, and computed tomography (CT) scan [11]. Hemophagocytic lymphohistiocytosis (HLH) is common at diagnosis in patients with rapidly progressive infantile-onset LAL-D [12,13,14,15,16]. HLH is due to uncontrolled immune activation with an acute and rapidly progressive systemic inflammatory response. The accumulated lipids activate macrophages, leading to their transformation into foam cells. The activated macrophages produce proinflammatory cytokines, which contribute to systemic inflammation [17]. If specific treatment is not initiated in a timely manner, the prognosis of patients with HLH and underlying infantile-onset LAL-D is severe or even fatal [12,15,18].

### 3.2. Later-Onset LAL-D

Patients with later-onset LAL-D can present with a remarkably diverse range of phenotypes, ranging from the onset of symptoms in infancy to a diagnosis as late as age 80 years [19]. Given the nonspecific nature of the symptoms (Table 1), the disease is often misdiagnosed. In fact, many patients with later-onset LAL-D do not have clinical symptoms at diagnosis or have non-specific symptoms that may go unnoticed [10]. Therefore, clinical suspicion often arises from the presence of suggestive biochemical alterations. While hypertransaminasemia and lipid profile alterations are the main biochemical characteristics of LAL-D, they are not pathognomonic [10]. The lipid profile of LAL-D may be indistinguishable from that of other types of common genetic hypercholesterolemia such as familiar hypercholesterolemia (prevalence of 1:200 to 1:250), which is characterized by elevated LDL-cholesterol and reduced high-density lipoprotein (HDL) cholesterol [20]. The most characteristic clinical features suggestive of later-onset LAL-D include hepatosplenomegaly and hepatic steatosis, which can lead to fibrosis, cirrhosis, portal hypertension, and even hepatocarcinoma [21,22] (Table 1). Splenomegaly may be due not only to liver fibrosis/cirrhosis-driven portal hypertension, but also to massive infiltration of the spleen by foamy macrophages. Although later-onset LAL-D is diagnosed in both children and adults, some of the signs and symptoms, such as hepatosplenomegaly, may not appear until older age [19]. Moreover, because of persistent dyslipidemia, patients with later-onset LAL-D are at risk for accelerated atherosclerosis and premature cardiovascular disease [2]. In later-onset LAL-D, calcification of the adrenal glands is not frequent.

## 4. Diagnosis

As the prevalence of LAL-D is low and the symptoms are non-specific, diagnosis can be challenging, leading to underdiagnosis [4]. Diagnosis is based on physical and clinical evaluation, biochemical analysis (especially liver and lipid profile), determination of LAL activity, and a genetic study of the *LIPA* gene.

### 4.1. Biochemical Suspicion

The most consistent biochemical abnormalities in LAL-D are elevated levels of liver enzymes, elevated LDL-cholesterol, and low HDL-cholesterol [23] (Table 1).

Three biochemical phenotypes can be distinguished: that characterized by hepatic impairment, that dominated by dyslipidemia, and the mixed phenotype. Patients do not always present hepatic and lipid alterations, as neither may appear at onset but rather become apparent as the disease progresses. In some instances, LAL-D can mimic pure dyslipidemias, such as heterozygous familial hypercholesterolemia and familial combined hyperlipidemia. LAL-D should be ruled out in patients with suspected heterozygous familial hypercholesterolemia not confirmed by genetic testing, particularly in those not responding adequately to statins [24,25,26].

High ferritin values and fat-soluble vitamin deficiencies (vitamins A, D, E, and K) are common at diagnosis in infantile-onset LAL-D [3].

When infantile-onset LAL-D co-occurs with HLH, other biochemical features can overlap with the biochemical alterations usually observed in LAL-D, rendering diagnosis even more challenging. The most common alterations observed in HLH are as follows: cytopenia in more than two cell lineages; elevated ferritin, triglycerides, liver enzymes, bilirubin, lactate dehydrogenase, and soluble IL-2Ra/CD25 (>2400 IU/mL); low fibrinogen; and low or absent NK cytotoxic activity. The presence of hemophagocytosis in the bone marrow also supports the diagnosis of HLH [17,27].

**Figure 2:**
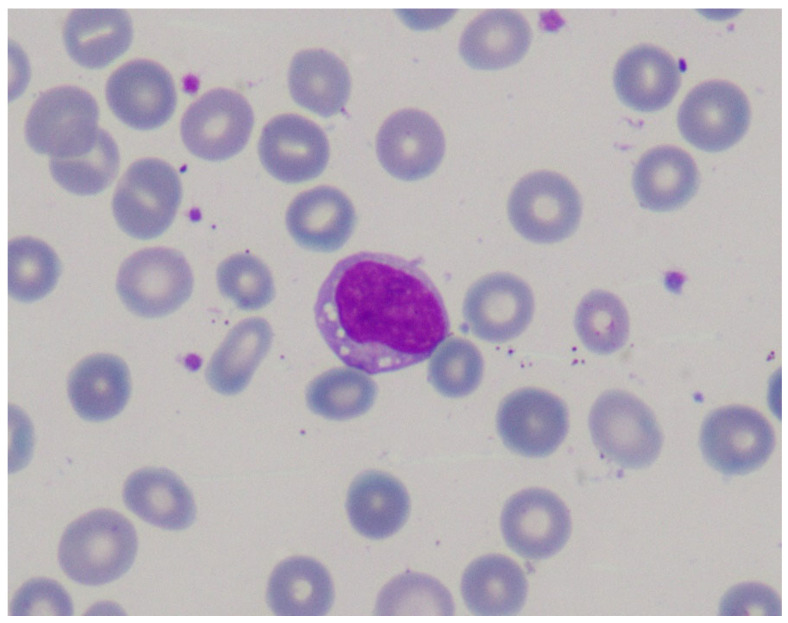
Blood film examination showing lymphocytes with cytoplasmatic lipid vacuoles in a patient with infantile-onset lysosomal acid lipase deficiency (LAL-D). Image courtesy of Dr. de las Heras.

Lymphocytes with cytoplasmatic lipid vacuoles can be detected in peripheral blood using a very rapid method from a simple blood sample by hematoxylin–eosin staining [28] (Figure 2). As in other lysosomal diseases, blood film examination for vacuolated lymphocytes in LAL-D constitutes an inexpensive, rapid, and minimally invasive clue to diagnosis. Notably, the absence of vacuolated lymphocytes does not rule out the diagnosis of LAL-D.

### 4.2. Determination of LAL Activity

In rapidly progressive infantile-onset LAL-D, LAL activity is close to zero. In the case of the later-onset phenotype, enzyme activity can vary (with 1–12% of activity observed in healthy individuals) [2]. Determination of the activity of LAL is a reliable and reproducible procedure that can be carried out on various biological samples, such as dry blood spot samples, peripheral blood leukocytes, and fibroblasts [29,30].

Analysis of LAL from a dried blood spot sample enables indirect quantification of LAL activity. Although it can successfully differentiate patients with LAL-D from healthy individuals, it cannot differentiate between a sample from a patient with infantile-onset LAL-D and a sample from a patient with later-onset LAL-D. Dried blood spot testing is easy and rapid and can be carried out at the slightest suspicion of the disease. LAL activity is determined in the presence of substrates (such as palmitic acid) covalently modified with a fluorochrome (4-methylumbelliferone). Enzyme activity leads to the release of the fluorescent compound, which can be quantified using fluorescence emission spectroscopy. Given that many lipases can be identified and that non-specific LAL substrates are used, testing is with a potent, highly specific LAL inhibitor (Lalistat-2) to ensure specificity. Enzyme activity, which is determined based on the difference between the sample incubated with and without the inhibitor [29], makes it possible to classify patients and thus rule out the disease when LAL activity is within or above the normal range (0.59–2.40 nmol/punch/h). Enzyme activity below the normal range necessitates the exclusion of possible anomalies in steps before the determination, such as inadequate sample collection or preservation. In such cases, other lysosomal reference enzymes (for example β-galactosidase) are determined [10,29,31]. If the activity of the reference lysosomal enzyme is within the normal range, an analysis of the *LIPA* gene should be performed. In order to obviate the need for two assays performed in parallel, i.e., one in the presence of an LAL-specific covalent inhibitor, Lalistat-2, and one to calculate the activity of LAL based on the difference between the two assays, a new test has been developed based on an ester formed between palmitic acid and 4-propyl-8-methyl-8-hydroxycoumarin [32]. The selectivity of this substrate for LAL is greater than 98%, leading to an approximately two-fold increase in the specific activity of LAL compared with the previously reported LAL assay [32]. Thus, patients with LAL-D can be readily identified with the new LAL assay using mass spectrometry or fluorometric assay platforms.

### 4.3. Molecular Analysis of the LIPA Gene

The *LIPA* gene is located on chromosome 10q23.2-q23.3 and consists of 10 exons distributed along 36 kb [33]. There are more than 600 variants reported in the ClinVar database and more than 2000 in Mastermind (Genomenon), although most are variants of unknown significance. Among these, more than 100 variants are classified as pathogenic or likely pathogenic (n = 127, ClinVar consulted on 18 March 2024) and are considered disease-causing variants (around 25% of them associated with the infantile-onset phenotype, 50% with the later-onset phenotype, and 25% with both). Most patients with LAL-D have single-nucleotide variants or rearrangements of the coding region of the *LIPA* gene. Infantile-onset LAL-D is associated with a higher proportion of frameshift and premature stop variants than later-onset LAL-D. However, when pathogenic variants are located in the intronic or regulatory regions of the gene, they are not detected using classic analyses, e.g., exome sequencing, and additional approaches such as transcriptome and/or whole-genome sequencing are needed to diagnose affected patients [10]. Therefore, in such cases, it may be difficult to confirm the molecular diagnosis. In the case of a patient with suspected LAL-D and low enzyme activity but inconclusive findings in the genetic study, the patient should be diagnosed with LAL-D.

The *LIPA* pathogenic variant most commonly associated with later-onset LAL-D is the nucleotide change c.894G>A, which affects the last nucleotide of exon 8 without changing the amino acid (p.Q298Q), although it does lead to the skipping of exon 8, which causes an in-frame deletion of 24 amino acids (p.S275_Q298del) [34]. This splice-junction variant in exon 8 (rs116928232: NM_000235.4:c.894G>A, NP_000226.2:p.S275_Q298del) is referred to as E8SJM [8]. According to a study performed in more than 15,000 *LIPA* alleles from healthy African American, Asian, Caucasian, Hispanic, and Ashkenazi Jewish individuals from New York and Dallas, the frequency of the c.894G>A allele was 1:1000 for the Asian population and approximately 1:300 for the Caucasian and Hispanic populations. No African American heterozygotes were detected. Assuming that c.894G>A accounted for 60% of reported multi-ethnic later-onset LAL-D variants, the predicted prevalence of later-onset LAL-D is approximately 0.8 per 100,000 [8]. This predicted prevalence is higher than observed in clinical practice, indicating that LAL-D is under-diagnosed and may be unmasked in patients presenting other, more common diseases, such as non-alcoholic fatty liver disease (NAFLD) and heterozygous familiar hypercholesterolemia [8].

A study of 23 Spanish patients with LAL-D (13 with infantile-onset disease and 10 with later-onset disease) revealed the novel c.966+2T>G variant, which accounted for 75% of the infantile-onset LAL-D alleles, and the frequent later-onset–associated c.894G>A variant, which accounted for 55% of the later-onset LAL-D alleles [35].

### 4.4. Other Diagnostic Tests

#### 4.4.1. Biomarkers

While plasma biomarkers, including liver enzymes and lipid profiles (such as ALT, AST, LDL-cholesterol, and HDL-cholesterol), are routinely used in the management of LAL-D, a disease-specific biomarker has yet to be identified. Although LAL-D primarily leads to the accumulation of cholesterol esters and triglycerides due to impaired lipid metabolism, this buildup also induces secondary processes such as inflammation and oxidative stress [36]. Regarding inflammation, patients with LAL-D have higher plasma chitotriosidase activity and higher chemokine (C-C motif) ligand 18/pulmonary and activation-regulated chemokine (CCL18/PARC) concentrations [36,37]. These biomarkers are released by activated macrophages and are elevated in other lysosomal diseases such as Gaucher disease, acid sphingomyelinase deficiency (ASMD), and Niemann–Pick disease type C (NPC), as well as in other conditions such as atherosclerosis [37,38,39,40,41]. It is advisable to study the gene that encodes chitotriosidase (*CHIT1*, MIM*600031), because patients may present reduced or zero activity of this enzyme owing to the presence of a 24–base pair duplication variant in heterozygosity or homozygosity, respectively [42]. While not specific for LAL-D, these biomarkers are useful for identifying patients with this condition [43].

Other biomarkers with elevated levels in the plasma of affected patients include cholesterol oxidation products known as oxysterols [36,37,44], such as 7-ketocholesterol and cholestane-3β,5α,6β-triol, which are markers of oxidative stress [45]. Oxidative stress, driven by an imbalance between reactive oxygen species and antioxidant defenses [46], may contribute to the progression of LAL-D by promoting cellular dysfunction and the onset of clinical manifestations. These oxysterols are also elevated in other diseases, such as ASMD, and, specifically, in conditions with alterations in cholesterol metabolism, such as NPC [37,44]. Additional studies are required to evaluate the potential of these biomarkers for patient monitoring and to identify disease-specific biomarkers.

#### 4.4.2. Diagnostic Tools: Imaging, Biopsy, and Histology

Abdominal X-ray, ultrasound, and CT scan showing adrenal gland calcification (Figure 1) are strongly suggestive of LAL-D. This very characteristic feature of the disease results from necrosis of adrenocortical cells overloaded with hydrophobic lipids. Adrenal calcifications are present in around 50% of patients with infantile-onset LAL-D and have been reported in some patients with the later-onset phenotype [2]. The CT scan can also detect adrenal hypertrophy not observable in plain radiography, as well as hepatosplenomegaly and portal hypertension.

Magnetic resonance imaging is the gold standard for the quantification of hepatic fat [47]. However, liver ultrasound can be considered in children for whom sedation is to be avoided.

Given the limitations of liver biopsy, numerous non-invasive tests have been developed and have proven accurate for the assessment of NAFLD-related fibrosis. Although the most frequently used method is transient elastography (TE), other common techniques include two-dimensional shear wave elastography and magnetic resonance elastography. According to the experts, caution must be exercised when extrapolating the results of these techniques obtained in other entities (especially TE and shear wave). The behavior of the techniques may differ, as fibrosis could be underestimated in LAL-D owing to the massive infiltration of fat in the liver. This uncertainty could be addressed in further studies, including serial analysis of individual patients over time with liver biopsy.

Biopsy of the liver, intestine, adrenal glands, and other organs can prove useful for diagnosis. Given its invasive nature and the availability of other non-invasive, rapid, and reliable methods, biopsy should be applied on an individual basis. Even so, liver biopsy is the gold standard for the evaluation of liver involvement and assessment of fibrosis [10]. In fact, up to two thirds of patients with LAL-D have liver fibrosis/cirrhosis [2]. Macroscopically, the liver of affected patients is yellow-orange. Microscopically, histopathology of liver samples from patients with LAL-D reveals an accumulation of cholesterol esters and triglycerides in the lysosomes of hepatocytes and Kupffer cells, often resulting in microvesicular steatosis and micronodular cirrhosis, in contrast to the large fat vacuoles displacing the nucleus to the periphery in macrovesicular steatosis. The observation of massive microvesicular steatosis on liver biopsy strongly suggests a lysosomal disease, especially LAL-D [37,48]. In addition, the accumulation of cholesterol esters seen as birefringent crystals in polarized light are pathognomonic. Typically, birefringence disappears when the sample is heated to 50–60 °C and reappears when the sample is cooled [49,50].

Lysosomal markers can be stained through immunohistochemistry in paraffin-embedded liver specimens to distinguish lipid accumulation in the cytosol or in the lysosomes and thus facilitate the diagnosis of LAL-D. These markers include cathepsin D, lysosomal-associated membrane protein (LAMP) 1, LAMP 2, and lysosomal integral membrane protein 2 [49]. The macroscopic appearance of the intestine (both small and large) is pale, with less thickness and a smaller number of mucosal folds. Microscopically, the presence of histiocytes with cytoplasmic lipid-laden vacuoles throughout the lamina propria is detected [51]. The adrenal glands are enlarged and yellowish in appearance, with dotted or clustered calcifications in areas of necrosis. Calcifications are common in the infantile-onset form but not in the later-onset phenotype. Biopsy of other specimens from other sites, such as the spleen, lymph nodes, bone marrow, and peripheral blood lymphocytes, may reveal characteristic lipid-filled cytoplasmic vacuoles.

Enlarged mesenteric lymph nodes have been associated with enteral administration of lipids. Biopsy to rule out malignancy has been performed in several patients, all of whom had foamy macrophages [52,53].

### 4.5. Differential Diagnosis

The symptoms observed in patients with infantile-onset LAL-D (e.g., hepato-splenomegaly, hypertransaminasemia, and hypercholesterolemia) could lead the condition to be confused with a glycogen storage disease, ASMD, NPC, Gaucher disease, bile acid synthesis defects, or HLH [10].

The lipid profile detected in patients with LAL-D is similar to that seen in other lipid metabolism disorders, such as heterozygous familial hypercholesterolemia, autosomal recessive hypercholesterolemia, familial combined hyperlipidemia, and sitosterolemia. The differential diagnosis is based on a detailed analysis of the family history and the hereditary pattern, differentiating between autosomal recessive disorders (LAL-D, autosomal recessive hypercholesterolemia and sitosterolemia) and autosomal dominant disorders (familial hypercholesterolemia). Because of the overlap in these entities, an expert panel of the European Arteriosclerosis Society has recommended a gene panel including *LDLR*, *apoB*, *PCSK9*, *LDLRAP1*, *ABCG5*, and *ABCG8*, as well as *LIPA*, in the diagnosis of primary hypercholesterolemia (Table 2) [54].

Regarding liver involvement, hepatomegaly, steatosis, and hypertransaminasemia are common in other, more frequent pathologies, such as NAFLD or cryptogenic cirrhosis [2,10]. The steatosis observed in patients with LAL-D differs from that observed in patients with NAFLD in that LAL-D patients have microvesicular steatosis, while NAFLD patients have macrovesicular steatosis (Table 2) [5,10,48].

Importantly, while overweight is not usual in LAL-D patients [2], it does not directly rule out the possibility of LAL-D.

Diagnosis of NAFLD is a diagnosis of exclusion. According to clinical guidelines, before the diagnosis can be confirmed, entities such as Wilson disease and autoimmune hepatitis must be ruled out [10,55]. LAL enzyme activity analysis could be included in this test series, and although liver biopsy is not widespread, clinical guidelines also recommend performing this investigation to establish the diagnosis of NAFLD and to assess the degree of fibrosis. LAL enzyme activity should be analyzed to rule out LAL-D, especially in cases involving microvesicular steatosis [10,55].

Vacuoles may also be present in foamy histiocytes and hepatocytes in systemic infections, cases of drug ingestion, hepatotoxins, and tumors (non-Langerhans xanthogranulomatous cell histiocytosis, inflammatory pseudotumor of the liver), as well as in other lysosomal storage diseases (ASMD, NPC, Gaucher disease, various forms of mucopolysaccharidosis, GM1 gangliosidosis, sialidosis) [56].

## 5. Treatment

The two pillars of treatment for LAL-D patients are a low-lipid diet and ERT with sebelipase α, an enzyme produced by recombinant DNA [57]. This agent was approved in Europe in 2015 for long-term ERT in patients with LAL-D of all ages and is aimed at restoring lost LAL activity physiologically by exogenous administration of a genetically engineered enzyme. The objective of a low-lipid diet is to reduce the substrate that leads to increased lysosomal, cell, and tissue deposition. Other therapeutic strategies include lipid-lowering therapy and hematopoietic stem cell or liver transplantation.

### 5.1. Infantile-Onset LAL-D (Wolman Disease)

Clinical onset of rapidly progressive infantile-onset LAL-D is a medical emergency. If specific treatment is not initiated in a timely manner, death typically occurs during the first six months of life [3]. Therefore, prompt diagnosis and urgent treatment are crucial, as ERT should be initiated as soon as possible, ideally before 2–3 months of age [7].

Once clinically suspected, LAL activity should be determined immediately, and the results should be made available as soon as possible, ideally within a few days, to ensure early initiation of appropriate treatment [4]. If clinical/biochemical suspicion is very high (e.g., an infant in whom typical clinical and biochemical findings co-occur with vacuolated lymphocytes in the blood film), we suggest initiating appropriate administrative procedures (e.g., urgent request to the pharmacy committee that approves special treatments in rare diseases) so that specific treatment can be started as soon as the diagnosis is established.

Careful nutritional management of affected patients with a low-lipid diet, along with prompt initiation of ERT, is essential [7]. In addition, infusions of sebelipase α must be initiated at a higher dosage than in later-onset LAL-D [57].

#### 5.1.1. Enzyme Replacement Therapy

The efficacy of sebelipase α in patients with infantile-onset LAL-D was assessed in LAL-CL03, a multicenter, open-label, phase 2/3 study conducted in nine countries. The study population comprised nine infants with growth failure before the age of six months [58]. Patients received once-weekly doses of sebelipase α, initiated at 0.35 mg/kg and increasing to 5 mg/kg. The main outcome measure was survival (at age 12 months and >24 months). Because ethical restrictions prevented the trial from being performed with a control group, treated patients were compared with a historical cohort of 21 untreated infants with LAL-D and similar clinical characteristics [58]. The results were found to show that 67% (95% CI, 30% to 93%) of sebelipase α-treated infants survived to 12 months of age compared with 0% (95% CI, 0% to 16%) of the historical control group. Improvements were recorded in patients who survived to age 12 months with respect to weight-for-age, reduced levels for the markers of liver dysfunction and hepatosplenomegaly, and improvements in anemia and gastrointestinal symptoms. Five of the nine infants who started the study survived beyond two years of age. Most deaths (three of four) occurred at the start of treatment, with ≤4 doses of sebelipase α, thus highlighting the importance of the early initiation of treatment. Treatment was generally well tolerated and, although half of the patients experienced some infusion-associated reactions, these were successfully managed following the usual protocols. No episodes of anaphylaxis were recorded, and treatment did not have to be discontinued in any patient [58].

An additional clinical study, LAL-CL08, and the follow-up of LAL-CL03 VITAL were analyzed, with the results both being reported jointly. Eligible participants received sebelipase α for up to three years (LAL-CL08) or five years (LAL-CL03 VITAL) [9]. The results from both trials (baseline to study end) revealed increases in median weight-for-age, length-for-age, and mid-upper arm circumference-for-age z scores and decreases in median liver and spleen volume. Values recorded for short-term transfusion-free hemoglobin returned to normal levels after a median of 4.6 months in all patients eligible for assessment in VITAL and after a median of 5.5 months in 70% of patients in LAL-CL08. Notably, overall survival was better in the LAL-CL08 study, though the trial recruited patients with more severe disease at onset. Kaplan–Meier survival estimates were 67% (to 12 months) and 56% (to 4 years) in VITAL, compared with 90% (to 12 months) and 80% (to 3 years) in LAL-CL08. Compared with the findings from VITAL, the increased survival in the LAL-CL08 study may be related to the higher initial and maximal doses of ERT, with faster dose escalation making it possible to maintain the same frequency as a weekly administration. Six of the nineteen patients that were analyzed died, though no deaths were found to be treatment related. Four of the deceased patients had received ≤4 doses of sebelipase α [9]. The efficacy and safety profile of sebelipase α remained unchanged in this group of patients after a long-term follow-up of 10 years [59,60,61].

Although no clear relationship was observed between the appearance of anti-drug antibodies (ADA) and the efficacy or safety of the drug in LAL-CL03 [58], the development of significant neutralizing ADA associated with an attenuated response to ERT was reported in three children with complete deletion of the *LIPA* gene [62]. Thus, patients with complete deletion of the *LIPA* gene may have a higher risk of developing a significant neutralizing ADA response, similar to cross-reactive immunological material-negative Pompe disease patients, and may benefit from a prophylactic immune tolerance induction regimen [63].

#### 5.1.2. Nutrition

LAL-D is a lysosomal storage disease characterized by the accumulation of lipids (cholesterol esters and triglycerides) that should be managed with a low-lipid diet as a substrate reduction therapy. At diagnosis, infantile-onset LAL-D almost invariably involves gut damage due to the accumulation of lipids along the lamina propria, leading to malabsorption and profound failure to thrive. As a result of gut damage, most infants develop protein intolerance and only tolerate elemental formulas.

Given that most commercially available elemental formulas are rich in lipids and long-chain triglycerides, the most suitable formula for infants with infantile-onset LAL-D is almost always a bespoke low-lipid elemental modular formula [64]. Table 3 shows a practical example of a low-lipid elemental modular formula for a patient recently diagnosed with infantile-onset LAL-D. This formula should be based on minimal fat, amino acids, and monosaccharides. In this low-lipid diet, the recommended intake of essential fatty acids and long-chain polyunsaturated fatty acids should be ensured, and medium-chain triglycerides (up to 1 g/kg/day) should be used to provide energy.

Affected patients have an increased protein requirement owing to digestive losses. Therefore, a minimum protein intake of 4 g/kg/day should be provided in the form of free amino acids. Glucose polymers can be used as carbohydrates and should be gradually increased up to 20% of total energy requirements or according to tolerance. In addition to macronutrients, it is important to ensure an adequate supply of electrolytes, vitamins (fat-soluble and water-soluble), and liquids tailored to the age and needs of the individual patient. In this case, multivitamin, mineral, and trace element preparations (e.g., Paediatric Seravit^®^) can be used, as can oral rehydration solutions. Due to malabsorption and organomegaly, enteral feeding may initially be better tolerated through a nasogastric tube in small boluses or as a continuous infusion.

Most infants with infantile-onset LAL-D require a period of parenteral nutrition along with modular enteral feeding until gastrointestinal symptoms subside and total enteral feeding can be administered. This should be a low-lipid parenteral nutrition (≤1 g lipid/kg/day using a lipid source containing medium-chain triglycerides, e.g., SMOFlipid) with high protein content (4 g/kg/day) and glucose content (12 g/kg/day up to 25 g/kg/day) to ensure sufficient calories to overcome malnutrition. Blood glucose levels should be monitored while the patient is receiving high-glucose parenteral nutrition [65]. In most cases, establishing total enteral feeding can take up to 1–6 months from the start of ERT, which should combine enteral and parenteral nutrition. Enteral feeds may be complicated by recurrent vomiting and diarrhea. However, unless they limit growth or cause significant weight loss due to dehydration, enteral feeds can continue to be used.

After some time with elemental modular formula, and once gut damage has been restored, whole protein feeds can be gradually introduced over 4–6 weeks using a low-fat formula (e.g., Monogen^®^, Lipistart^®^). In patients who initiate treatment early and progress favorably, the switch from a low-lipid elemental modular formula to a low-fat formula with whole protein could be made in one day.

Given that weight and body mass index may not be indicative of nutritional status because of the influence of factors such as organomegaly and ascites, mid-upper arm circumference should be monitored.

It is also important to monitor circulating lipid-soluble vitamins, essential fatty acids, and iron levels in affected patients. Supplements of each should be provided if necessary. Intravenous iron supplementation may be required.

#### 5.1.3. Hematopoietic Stem Cell Transplantation

Before ERT became available, the results of hematopoietic stem cell transplantation were unsatisfactory: morbidity and mortality were very high, possibly because pathophysiology is predominantly mediated by deficient enzyme activity in bone marrow-derived monocyte macrophages [66,67]. More recently, a retrospective analysis evaluated 24 children with infantile-onset LAL-D who underwent hematopoietic stem cell transplantation. Five patients (21%) had previously received ERT. Administration of ERT with fludarabine, treosulfan, and thiotepa improved transplant survival for infants with LAL-D. Analysis according to pre-transplant ERT revealed a five-year overall survival of 80% (95% CI, 20% to 97%) for children who received ERT and 21% (95% CI, 7% to 41%) for children who did not (*p* = 0.03) [68]. In the five patients with infantile-onset LAL-D who received ERT before transplantation, the combination of ERT with reduced dietary substrate stabilized the sick infant and reduced transplant-associated mortality [62]. ERT with reduced dietary substrate before transplant has been proposed as an alternative for the treatment of infantile-onset LAL-D [62,69]. However, mixed chimerism is frequent after hematopoietic stem cell transplant, indicating an engraftment defect. Long-term data on patients who have received this regimen have not yet been reported.

#### 5.1.4. Initial Approach and Specific Problems in Infantile-Onset LAL-D

ERT is effective in infantile-onset LAL-D if initiated early. Therefore, once the disease is suspected, LAL enzyme activity should be determined immediately in a dried blood spot sample. Once the diagnosis is confirmed, a low-lipid diet and ERT with sebelipase α should be started urgently at an initial dose of 3 mg/kg/week, increasing to 5 mg/kg/week in cases of suboptimal clinical response [57]. Recent reports point to good progress when treatment is started at higher doses, up to 5 mg/kg twice a week [70].

If the patient develops HLH, red blood cell concentrate transfusions and anti-inflammatory treatment may be needed.

Importantly, despite the early initiation of specific treatment (at around two months of life or earlier), the first months to one year of life can be challenging, necessitating very careful management.

After initiation of ERT, rapid improvement of biochemical parameters and hepatosplenomegaly are common. However, gut damage takes longer to recover. Malabsorption, vomiting, and diarrhea leading to malnutrition may persist, and careful nutritional management is necessary to maintain the child’s nutritional status. Because of gut damage, infants often present protein intolerance, and a bespoke modular low-lipid elemental formula is usually needed (Table 3). For a period of time—usually some months—patients typically require a combination of enteral nutrition combined with low-lipid parenteral nutrition until gut damage recovers and complete enteral nutrition can be administered.

Consideration should be given to periods of intercurrent illness, such as viral or central catheter infections, in which digestive symptoms can be exacerbated, leading to significant weight loss. Therefore, intercurrent processes should be managed with particular attention to calorie intake, usually requiring the use of a nasogastric tube. Intercurrent non-adherence to diet may lead to a systemic inflammatory response.

### 5.2. Later-Onset LAL-D

In patients with later-onset LAL-D, a low-lipid diet is recommended as substrate reduction therapy, although a strict low-lipid diet is not usually necessary. The recommended dosage of sebelipase α is 1 mg/kg, administered as an intravenous infusion once every other week, with dose escalation to 3 mg/kg once every other week in cases of suboptimal response based on clinical and/or biochemical criteria [57]. Lipid-lowering agents, especially ezetimibe, may also play a role in the treatment of later-onset LAL-D, although they have no effect on the progression of liver histology findings [22].

#### 5.2.1. Enzyme Replacement Therapy

LAL-CL02 (ARISE study) evaluated the efficacy of sebelipase α in patients with later-onset LAL-D [71,72]. This phase 3, multicenter, double-blind study enrolled 66 patients aged >4 years who were randomized to receive sebelipase α (n = 36) or placebo (n = 30) for 20 weeks. All patients then had the option of being included in an open-label phase with sebelipase α. At 20 weeks, normal alanine aminotransferase (ALT) levels were detected in 11 patients (31%) in the sebelipase α group and in two (7%) in the placebo group (*p* = 0.03). The decrease in the mean ALT level from baseline was significantly greater in the sebelipase α group than in the placebo group (−58 U/l vs. −7 U/l, *p* < 0.001) [71]. At 256 weeks (study end), ALT values had returned to normal in 47% of patients. Among patients with baseline ALT levels ≥ ×3 upper limit of normal, ALT values returned to normal in 49% [72]. Among patients receiving ERT, significant improvements were observed compared with placebo at week 20 in LDL-cholesterol (−28% vs. −6%; *p* < 0.001), non-HDL-cholesterol (−28% vs. −7%; *p* < 0.001), HDL-cholesterol (20% vs. −0.3%; *p* < 0.001), and triglyceride levels (−26% vs. −11%; *p* = 0.04). The number of patients with adverse events was similar in the treatment and control groups, with most events being classified as mild [71,72]. After 52 weeks of sebelipase α, changes in the Ishak stage from baseline showed that 92% of patients with paired biopsy data had an improved or stable Ishak stage, with a ≥1-stage improvement in 67% and a ≥2-stage reduction in 50% [72].

A subsequent subanalysis of this study to assess the effects of sebelipase α on atherogenic biomarker levels [73] showed a significant improvement in altered lipid profiles, independent of the use of lipid-lowering agents. The efficacy and safety profile of sebelipase α remained unchanged after a long-term follow-up of five years [72].

#### 5.2.2. Lipid-Lowering Drugs

As ERT improves the lipid profile [7], concomitant use of lipid-lowering drugs is not usually required in LAL-D patients receiving ERT.

Statins have been administered in patients with LAL-D, even as long-term therapy, although they are a controversial agent and unlikely to be beneficial in this population [1,2,74,75]. Statins could potentiate the disease mechanism, as inhibiting 3-hydroxy-3-methylglutaryl coenzyme A (HMG-CoA) reductase causes a decrease in free cholesterol synthesis, with an increase in uptake of LDL in the liver, providing more cholesteryl esters and triglycerides to the lysosomes. Accordingly, liver injury and fibrosis in patients with LAL-D often progress in the long term despite the administration of statins [10,76,77,78,79].

The lipid-lowering drug ezetimibe is also used in patients with LAL-D. Ezetimibe reduces absorption of cholesterol in the intestine by inhibiting the NPC1L1 transporter protein found in the intestinal microvilli. Studies in LAL-deficient mice have shown that ezetimibe reduces liver volume, hepatic cholesterol concentrations, and serum transaminases [80].

#### 5.2.3. Liver Transplantation

Bernstein et al. [81] analyzed the progress of 18 patients with LAL-D after liver transplantation, reporting a high percentage of recurrence (61%) and mortality (33%). The high frequency of recurrence was probably due to macrophages re-infiltrating the transplanted liver.

There are very few reported cases of patients with later-onset LAL-D and no signs of liver disease recurrence some years after transplantation [82]. Liver transplantation may be able to resolve the hepatic phenotype in some cases. However, the impact on the systemic accumulation of cholesterol esters is currently unknown.

### 5.3. Treatments Under Investigation

As has been reiterated in this manuscript, early initiation of treatment greatly determines prognosis in infantile-onset LAL-D. In this sense, a clinical trial is currently recruiting patients to study the safety and efficacy of in utero ERT in different lysosomal storage diseases, including LAL-D (clinicaltrials.gov, NCT04532047) [83].

Gene therapy is an interesting therapeutic approach, currently under investigation for lysosomal storage diseases [84]. Regarding LAL-D, a recent study has shown promising results for a single-treatment therapy using an adeno-associated virus in a preclinical model of Lipa-/- mice [85].

## 6. Proposed Diagnosis and Follow-Up Algorithms

**Figure 3:**
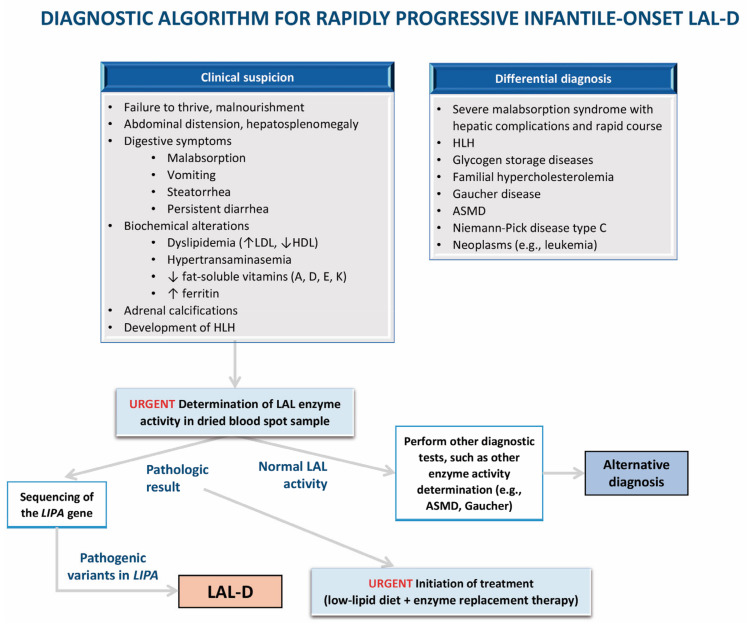
Algorithm for the diagnosis of infantile-onset lysosomal acid lipase deficiency (LAL-D). ASMD: acid sphingomyelinase deficiency; LAL: lysosomal acid lipase; LAL-D: lysosomal acid lipase deficiency; HDL: high-density lipoprotein; LDL: low-density lipoprotein; HLH: hemophagocytic lymphohistiocytosis.

**Figure 4:**
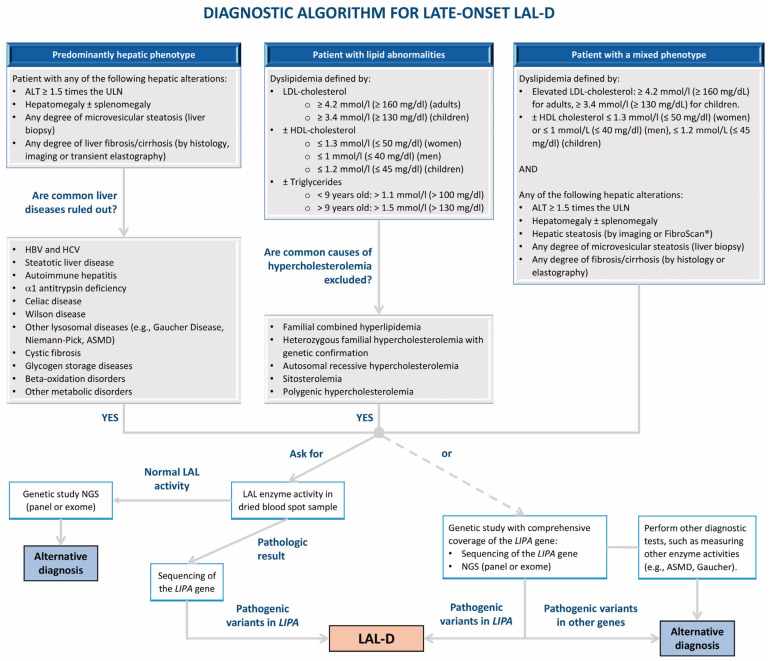
Algorithm for the diagnosis of later-onset lysosomal acid lipase deficiency (LAL-D). ALT: alanine aminotransaminase; ASMD: acid sphingomyelinase deficiency; HBV: hepatitis B virus; HCV: hepatitis C virus; HDL: high-density lipoprotein; LAL: lysosomal acid lipase; LAL-D: lysosomal acid lipase deficiency; LDL: low-density lipoprotein; NGS: next-generation sequencing; ULN: upper limit of normal.

Figure 3 and Figure 4 show proposed algorithms for the diagnosis of infantile-onset and later-onset LAL-D, respectively. Table 4 shows follow-up recommendations for LAL-D patients.

## 7. Transition from Pediatric to Adult Medicine

In some childhood conditions, such as diabetes, hereditary metabolic diseases, and rare liver diseases, increasing attention has been paid to the need to support patients in their transition from pediatric to adult care. In the case of patients with LAL-D, this process must be carefully planned, with every effort being made to focus on the specific needs of the patient. Planning requires close collaboration between the pediatric and adult medical teams to ensure that young people with LAL-D receive optimal care during this period. A recent survey of patients with lysosomal storage diseases [86] found that most patients (80%) knew they were being transferred to adult care, though half were not informed until after their 18th birthday. The authors stressed that most patients (>90%) valued three specific actions: access to explanatory documents, the transmission of their medical records to the adults’ section, and a joint consultation with both the pediatrician and the adult physician. Therefore, we must ensure that patients are fully informed about their disease and that their physicians are available for any disease-related query or clarification. The transition period is also an ideal time to inform the patient about the hereditary nature of LAL-D and the possible risks associated with reproduction and treatment during pregnancy. In summary, the transition is a process that should be considered as more than a simple bureaucratic transfer of information. It is essential that health professionals prioritize the patients’ needs and ensure that they feel empowered with respect to the management of their disease.

## 8. Strengths and Limitations

The major strength of this narrative review is that it was written by physicians with experience treating LAL-D patients (both the infantile-onset and the later-onset phenotypes) and clinical laboratory specialists knowledgeable in the condition. Furthermore, in this study we give voice to patients affected by LAL-D, with the participation of the president of the Spanish LAL-D Patient Organization. In addition, we provide practical information for the diagnosis, management, and follow-up of patients with LAL-D that we hope will be useful for physicians making new diagnoses in the future. The main limitation of this study is inherent to research in rare diseases. As LAL-D is an ultra-rare disease, much of the practical information provided in this manuscript does not come from studies with a high level of evidence, but rather from expert recommendations.

## 9. Conclusions

LAL-D is a severe and progressive ultra-rare disease for which effective treatment is available. When LAL-D is suspected, LAL enzyme activity should be determined in a dried blood spot sample. A low-lipid diet is an essential part of the treatment, as is ERT with sebelipase α. The clinical onset of rapidly progressive infantile-onset LAL-D is a medical emergency in which ERT and careful nutritional management should be initiated as soon as possible, as the prognosis is directly linked to the early initiation of specific treatment.

Survival in rapidly progressive infantile-onset LAL-D has improved dramatically with ERT. Furthermore, in recent years our knowledge of the management of these patients has increased considerably, with improvements regarding the initial ERT dose and careful nutritional treatment to decrease systemic inflammation, leading to better outcomes in the real world. Future research directions include in utero ERT, gene therapy, and therapeutic mRNA for intracellular protein replacement, all of which may fulfil currently unmet needs in the future. Genetic counseling is essential, and while newborn screening programs for the disease are not available at the time of writing, disease awareness programs could play an important role.

## Figures and Tables

**Table 1:** Clinical and biochemical manifestations in infantile-onset and later-onset lysosomal acid lipase deficiency.

Infantile-Onset LAL-D	Later-Onset LAL-D
Malabsorption Vomiting Diarrhea Malnourishment Failure to thrive Hepatosplenomegaly Calcification of the adrenal glands Anemia ↑ Hepatic transaminases ↑ LDL-, ↓ HDL-cholesterol ↓ Fat-soluble vitamins (A, D, E, K) Hemophagocytic lymphohistiocytosis	Liver microvesicular steatosis (which may progress to fibrosis, cirrhosis, and hepatocellular carcinoma) Hepatosplenomegaly ↑ Hepatic transaminases Mixed hyperlipidemia Hypoalphalipoproteinemia Premature atherosclerosis

LAL-D: lysosomal acid lipase deficiency; HDL: high-density lipoprotein; LDL: low-density lipoprotein.

**Table 2:** Differential diagnosis among LAL-D, familial hypercholesterolemia, and non-alcoholic fatty liver disease.

LAL-D	Familial Hypercholesterolemia	Non-Alcoholic Fatty Liver Disease
Autosomal recessive Biallelic *LIPA* mutation Raised liver enzymes High LDLc Low HDLc Variable increase in triglycerides Hepatomegaly Splenomegaly Microvesicular steatosis (liver biopsy) Variable degree of fibrosis usually present in pediatric population (liver biopsy)	Autosomal dominant Biallelic *LDLR*, *apoB*, *PCSK9*, *LDLRAP1*, *ABCG5*, and *ABCG8* mutations Normal liver enzymes High LDLc Normal HDLc No enlarged liver or spleen	Genetic or environmental origin Normal or raised liver enzymes Usually normal LDLc and HDLc No enlarged liver or spleen Macrovesicular steatosis (liver biopsy) Variable degree of fibrosis can be observed in adult population (liver biopsy)

HDLc: high-density lipoprotein cholesterol; LDLc: low-density lipoprotein cholesterol; LAL-D: lysosomal acid lipase deficiency.

**Table 3:** Practical example of a low-fat elemental modular formula for a patient with recently diagnosed infantile-onset lysosomal acid lipase deficiency (LAL-D).

Age: 2 Months of Life. Weight: 3.5 kg. An Enteral Low-Fat Elemental Modular Formula Is Initially Provided by Continuous Feeding at the Volume the Infant Tolerates Owing to Gut Damage, Malabsorption, and Abdominal Distension. Low-Lipid Parenteral Nutrition Combined with the Enteral Modular Formula in Most Cases.								
Initial Diet *	Product	Amount	Protein	Lipids	Carbohydrates	Energy (kcal)	Na	K
Proteins	Essential amino acid mix (Nutricia)	17.5 g (4 g/kg protein equivalent)	14 g	0	0	3.16 kcal/g	0	0
Glucose polymer	Fantomalt (Nutricia), Vitajoule (Vitaflo)	35 g ** (10% dextrinomaltose)	0	0	33.25 g	3.8 kcal/g	0	0
MCT oil	MCT oil (Nutricia)	3.5 mL (1 mL/kg)	0	3.3 g	0	8.55 kcal/ml	0	0
Vitamins and mineral supplement	Paediatric Seravit (Nutricia)	17.5 g (5 g/100 mL)	0	0	13.12 g	2.93 kcal/g	40 mg/ 100 g	30 mg/ 100 g
Essential Fatty acids	KeyOmega (Vitaflo)	4 g	0	0.8 g DHA 100 mg AA 200 mg	2.8 g	19 kcal per sachet	0	0
Ions ***	e.g., Bioralsuero Casen	180 mL	0	0	2.6 g	5.8 kcal/ 100 mL	146.4 mg/ 100 mL	78.6 mg/ 100 mL
Fluid	Water	To 100 mL/kg	0	0	0	0		

Nutritional information: 300.01 kcal (85.72 kcal/kg), fluids 350 mL, 0.86 kcal/mL; proteins 14 g, 56.04 kcal (18.68%); carbohydrates 51.9 g, 207.16 kcal (69.05%); fat 4 g, 36.81 kcal (12.27%); Na 286.52 mg (12.45 mmol → 3.5 mmol/kg); K 163.13 mg (4.1 mmol → 1.1 mmol/kg). * Liquids, ions, and vitamins must be adjusted according to the patient’s needs. As this is a modular diet, when administered in continuous infusion, it may frequently require shaking to prevent it from precipitating. ** We can start with dextrinomaltose at 10% and progressively increase the dextrinomaltose concentration up to 20%. For example, if the dextrinomaltose concentration is increased to 15% (52.5 g), the nutritional content of the modular formula would be as follows: 366.53 kcal (proteins 15.2%; lipids 10%; carbohydrates 74.6%), 104.72 kcal/kg, 1 kcal/mL. *** Depending on availability, the ion source can be different. Oral rehydration serum or ions at different concentrations may be used. AA: arachidonic acid; DHA: docosahexaenoic acid; LAL-D: lysosomal acid lipase deficiency; MCT: medium chain triglyceride.

**Table 4:** Follow-up schedule for patients with lysosomal acid lipase deficiency (LAL-D) starting treatment with sebelipase α.

Interventions	First Year						After First Year		
	Baseline ^a^	1 Month	3 Months	6 Months	9 Months	12 Months	Every 6 Months	Every 12 Months	Every 24 Months
**Recommended**									
Clinical evaluation ^b^	√	√	√	√	√	√	√		
Abdominal ultrasound	√					√		√	
Complete blood cell count ^c^	√	√ Infantile-onset	√ Infantile-onset	√ Infantile-onset		√		√	
Liver function ^d^	√	√	√	√	√	√	√		
Basic lipid profile ^e^	√	√	√	√	√	√	√		
Extended lipid profile ^f^	√					√		√	
Other biomarkers ^g^	√			√		√	√		
Iron metabolism	√	√ Infantile-onset	√ Infantile-onset	√ Infantile-onset		√		√	
Lipid-soluble vitamins (A, D, E, K)	√	√ Infantile-onset	√ Infantile-onset	√ Infantile-onset	√ Infantile-onset	√		√	
Polyunsaturated fatty acids	√			√ Infantile-onset		√		√	
Diet evaluation	√	√	√	√	√	√		√	
**Optional**									
Liver biopsy ^h^	√								Repeat in 2 years (non-periodic)
Fibroscan	√					√		If abnormal	If normal
MRI/liver ultrasound	√							If abnormal	If normal
Echocardiography	√							If abnormal	If normal
Carotid ultrasound ^i^	√							If abnormal	If normal
Fibrosis serological markers and scores ^j^	√					√		√	

√: Perform in both infantile- and later-onset LAL-D. √ Infantile-onset: perform only in infantile-onset LAL-D. ^a^ Treatment initiation with sebelipase α. ^b^ Evolution of symptoms and/or onset of new symptoms, anthropometry (weight, height, body mass index, brachial circumference), and physical examination. ^c^ Complete blood cell count, red blood cell count, white blood cell count, hemoglobin level, hematocrit, platelet count, international normalized ratio, and coagulation test. ^d^ Alanine aminotransferase, aspartate aminotransferase (AST), gamma glutamyl transferase, albumin, protein, total and conjugated bilirubin, alkaline phosphatase, lactate dehydrogenase. ^e^ Total cholesterol, low-density lipoprotein cholesterol, high-density lipoprotein cholesterol, and triglycerides. ^f^ Basic lipid profile + ApoA1, ApoB100, and lipoprotein(a). ^g^ Oxysterols, chitotriosidase, CCL18/PARC. ^h^ Liver biopsy is the reliable “gold standard” for assessing liver damage and monitoring disease progression. However, as it is an invasive method, its use must be assessed on an individual basis. ^i^ Intima-media thickness measurement. ^j^ According to local availability (e.g., AST-to-platelet ratio index or Forns index for prediction of liver fibrosis). This follow-up schedule was developed by the authors based on that of Camarena et al. [10].
